# Serum CA-125 for the diagnosis of pulmonary tuberculosis: a systematic review and meta-analysis

**DOI:** 10.1186/s12879-021-06772-7

**Published:** 2021-10-23

**Authors:** Ping Zhao, Qin Yu, Aijie Zhang, Fang He, Songyan Xu, Liang Chen

**Affiliations:** 1 Department of Clinical Laboratory, Beijing Shijitan Hospital, Capital Medical University, Beijing Key Laboratory of Urinary Cellular Molecular Diagnostics, NO. 10, Tie Yi Road, Yang Fang Dian, Haidian District, Beijing, 100038 China; 2 Tuberculosis Clinic, Chaoyang District Centre for Disease Control and Prevention, Chaoyang District, Beijing, China; 3 Department of Internal Medicine, Shuangqiao Hospital, Beijing, China; 4 Department of Medical Imaging, Beijing First Hospital of Integrated Chinese and Western Medicine, Beijing, China

**Keywords:** Tuberculosis, Diagnosis, Serology, CA-125, Infection, Meta-analysis

## Abstract

**Background:**

Pulmonary tuberculosis (PTB) remains the world’s deadliest infectious killer. Serum CA-125 test are useful in the diagnosis of PTB. Although studies on the relation between CA-125 and PTB have been reported, the specificity and sensitivity of serum CA-125 in diagnosing PTB vary widely among different studies. The present study was performed to evaluate the accuracy of CA-125 for the diagnosis of PTB via a meta-analysis of data obtained from previous studies.

**Methods:**

English and Chinese medical electronic databases were searched for eligible studies published up to February 2020. STATA software was used to obtain a pooled estimation of the diagnostic accuracy of CA-125 and analyze the heterogeneity of the recruited studies. Quality Assessment of Diagnostic Accuracy Studies-2 (QUADAS-2) was used to evaluate the quality of the obtained studies.

**Results:**

A total of 16 articles were included in this study. The pooled sensitivity and specificity of CA-125 were 0.85 [95% confidence interval (CI) 0.75–0.91] and 0.87 (95% CI 0.78–0.93), respectively. Moreover, the pooled positive likelihood ratio (LR+), negative likelihood ratio (LR−), and diagnostic odds ratio (DOR) of CA-125 were 6.65 (95% CI 3.62–12.20), 0.18 (95% CI 0.10–0.31), and 37.82 (95% CI 13.17–108.60), respectively. The area under the summary receiver operating characteristic curve (AUC) was 0.93.

**Conclusions:**

Taken together, the results indicate that serum CA-125 presents potential practical value for diagnosing PTB, but its clinical applicability must be further examined.

## Background

Tuberculosis (TB), an important infectious disease caused by *Mycobacterium tuberculosis* (*M.tb*), remains the world’s deadliest infectious killer; indeed, close to 30,000 new cases and over 4000 deaths from this disease are recorded worldwide each day [1]. Pulmonary TB (PTB) is the most prevalent clinical manifestation of TB. Thus, the early diagnosis of PTB is of paramount importance in efforts to treat and control the spread of the disease. Imaging examination, sputum smear microscopy, and culture are among the most commonly used methods for diagnosing PTB [2]. However, the sensitivity of these techniques is usually low and variable [3]. Moreover, sputum testing is impossible in cases with PTB who are unable to expectorate. Although some rapid molecular testing methods with higher sensitivity are used in regions with advanced laboratory facilities, these methods are costly and difficult to implement in resource-limited settings [2].

Rapid serological diagnostic approaches are essential and useful in the diagnosis of PTB. Although a number of serological tests, such as interferon gamma release assays (IGRAs) and *M.tb* antibody detection, have been developed for the accurate and rapid diagnosis of *M.tb* infection, the test results are not correlated with disease activity or treatment responses [4, 5]. Besides IGRAs and TB-Ab detection, CA-125 also has been used for PTB diagnosis [6–8]. CA-125 is a high-molecular weight glycoprotein expressed on mesothelial cells lining the pleura, peritoneum, and pericardium and epithelial cells of the endometrium and fallopian tubes [9]. Elevated serum CA-125 levels can be detected in some patients with malignant diseases involving the breasts, lungs, ovaries, colon, and pancreas, as well as in others with non-malignant diseases, such as endometriosis, uterine myomas, ovarian cysts, hepatic cirrhosis, pleural effusions, peritonitis, pancreatitis, and heart failure [8–12]. Although studies on the relation between CA-125 and PTB have been reported, some scholars believe that the results of these studies are biased toward patients with pleural effusions [13, 14]. In addition, the specificity and sensitivity of serum CA-125 in diagnosing PTB vary widely among different studies, and no multicenter study with a large sample size has yet been published to confirm the value of serum CA-125 in diagnosing PTB.

The aim of the present study is to evaluate the diagnostic performance of serum CA-125 in PTB patients via a systematic review and meta-analysis of data collected from previous studies.

## Methods

### Literature search and selection

The present study was conducted in accordance with the recommendations of the Cochrane Diagnostic Test Accuracy Working Group [15]. The PubMed, Web of Science, Embase, Chinese BioMedical databases, China National Knowledge Internet (CNKI), Wanfang Data and Cochrane Databases were searched by two researchers to screen for eligible studies published up to February 2020 by using the keywords “Tuberculosis”, “CA-125”, “Carbohydrate antigen 125”, “Cancer antigen 125”, “Tumor marker”, and “Cancer marker” as a combination of free text and thesaurus terms. The reference lists of the obtained articles were also searched for possible candidate studies. No language limits were applied. The inclusion criteria were as follows: studies that assessed the diagnostic accuracy of serum CA-125 for PTB diagnosis, included PTB and non-PTB participants, and reported true-positive, true-negative, false-positive, and false-negative rates; in the absence of this last criterion, the data in the original studies must be sufficient to enable the corresponding rates. Studies that failed to meet the inclusion criteria or lacked the essential information were excluded from the analyses.

### Data extraction and quality assessment

Data extraction and quality assessment of the original studies were independently performed by two reviewers. The following information was recorded: author, year of publication, language, country, age of participants, number of cases, CA-125 detection method, reference standard, study design, and CA-125 cut-off, sensitivity, specificity, true-positive, true-negative, false-positive, and false-negative rates for the diagnosis of PTB.

The modified version of quality assessment of diagnostic accuracy studies tool-2 (QUADAS-2) was used to assess the methodological quality of the recruited articles [16]. Four domains, namely, patient selection, index test, reference standard, and flow and timing, were considered to assess the risk of bias, and three domains, namely, patient selection, index test, and reference standard, were assessed based on applicability. In this study, some items for patient selection in the risk assessment of bias were revised; the items used to assess the methodological quality of the studies are listed in Table 1.

**Table 1:** The assessment items of methodological quality of studies

Judgments on Bias	
Patient selection	1. Was a consecutive or random sample of patients enrolled?
	2. Did the control group include other respiratory diseases?
	3. Did the study include appropriate exclusions?
Index test	1. Were the index test results interpreted without knowledge of the results of the reference standard?
	2. If a threshold was used, was it pre-specified?
Reference standard	1. Is the reference standards likely to correctly classify the target condition?
	2. Were the reference standard results interpreted without knowledge of the results of the index tests?
Flow and timing	1. Was there an appropriate interval between index test and reference standard?
	2. Did all patients receive the same reference standard?
	3. Were all patients included in the analysis?
Applicability	
Patient selection	Are there concerns that the included patients and setting do not match the review question?
Index test	Are there concerns that the index test, its conduct, or its interpretation differ from the review question?
Reference standard	Are there concerns that the target condition as defined by the reference standard does not match the question?

Discrepancies in article screening, data extraction, and quality assessment were resolved by a third reviewer through discussion or arbitration.

### Statistical analysis

RevMan 5.2 software was used to assess the methodological quality of the included studies. STATA 15.1 software was used for the statistical analyses and data pooling for sensitivity, specificity, positive likelihood ratio (LR+), negative likelihood ratio (LR−), and diagnostic odds ratio (DOR). The summary receiver operating characteristic (SROC) curve was used to detect the diagnostic performance of serum CA-125 in PTB patients. The heterogeneity of the recruited studies was analyzed using the means of a test for the Q statistic, with the extent of heterogeneity determined using the *I*^2^ index. Here, *I*^2^ values of 25%, 50%, and 75% were considered to indicate low, moderate, and high heterogeneity, respectively. Meta-regression analysis by country, language, age of participants, number of cases, CA-125 detection method, and QUADAS-2 items was performed to discover potential sources of heterogeneity. Sensitivity analysis was performed to test the robustness of the results by using two methods: Cook’s distance was used to identify strongly influential studies, and a scatter plot of the standardized predicted random effects was used to detect outliers [17]. The meta-analysis was repeated to test the robustness of the results after the exclusion of strongly influential studies and outliers. Deek’s funnel plot asymmetry test was used to evaluate publication bias [18]. A two-tailed P-value < 0.05 was considered to indicate significant difference.

## Results

### Literature search

**Fig. 1:**
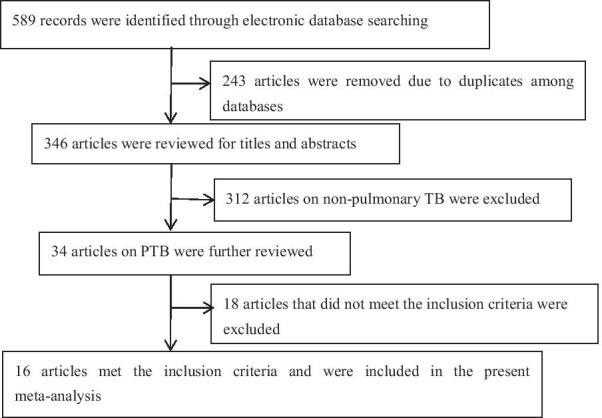
Flow chart of the process of the search strategy for study selection

A total of 589 articles (English articles: 334; French articles: 2; Japanese articles: 2; Chinese articles: 251) were screened from above-mentioned databases, and 243 duplicate articles were removed. The titles and abstracts of the remaining 346 articles were reviewed, and 312 articles on non-pulmonary TB were removed. The remaining 34 articles on PTB were further evaluated, and 18 articles that did not meet the inclusion criteria (they lacked some indicators, such as true-positive, false-positive, true-negative, and false-negative, or these indicators couldn’t be calculated from the data in the original studies) were excluded. Finally, 16 articles (English articles: 10; Chinese articles: 6) meeting the inclusion criteria were recruited in the study [6, 7, 19–32] (Fig. 1).

### Characteristics of the eligible studies

The characteristics of the 16 recruited studies are listed in Table 2. Six articles were published in Chinese from 2009 to 2019, and 10 articles were published in English from 2001 to 2018.

**Table 2:** Main characteristics of studies included in the meta-analysis

No.	Author	Country	Year	TP	FP	FN	TN	No. of Cases	Cut-off (U/ml)	Control	Golden standard	Language	Method	Age of cases	M/F
1	Du [14]	China	2014	100	10	19	26	119	35.00	Other respiratory disease	Clinical, radiological or bacteriological findings	Chinese	ECLIA	41 (22–78)	60/59
2	Du [7]	China	2017	87	2	15	46	102	13.60	Healthy individuals	Positive culture for Mtb in sputum	English	ECLIA	49.03 ± 3.23	77/25
3	Fortún [15]	Spain	2009	24	12	11	42	35	32.50	Other respiratory disease	Positive culture for Mtb in sputum or other tract respiratory samples	English	RIA	−	−
4	Liang [16]	China	2014	21	0	22	20	43	35.15	Heath individuals	Clinical, radiological or bacteriological findings	Chinese	ECLIA	45.27 ± 2.81	24/19
5	Liu [17]	China	2009	93	4	3	39	96	35.00	Inactive tuberculosis	Sputum smear positive for AFB or positive culture for Mtb	Chinese	ECLIA	52 ± 19	75/21
6	Ma [18]	China	2016	108	68	5	384	113	10.30	Heath individuals	Clinical, radiological and bacteriological findings	English	ECLIA	38.16 ± 18.86^§^; 52.18 ± 21.22^¶^	72/41
7	Mikačić [19]	Bosnia and Herzegovina	2017	30	59	10	121	40	35.00	Other respiratory disease	Positive culture for Mtb in sputum	English	CMIA	65 (18–89)^*^	163/57^#^
8	Mohammad [20]	Egypt	2016	33	11	7	29	40	21.05	Heath individuals and other respiratory disease	Clinical, radiological and bacteriological findings	English	ELISA	34.2 ± 15.15	35/5
9	Ozsahin [21]	Turkey	2008	19	48	11	68	30	35.00	Inactive tuberculosis and other respiratory disease	Sputum smear positive for AFB or positive culture for Mtb	English	MEIA	54 ± 15	−
10	Rizk [22]	Egypt	2018	40	5	2	22	42	37.60	Other respiratory disease	Clinical, radiological and bacteriological findings	English	ELISA	40.190 ± 13.095	33/9
11	Şahin [23]	Turkey	2012	41	0	1	55	42	36.35	Heath individuals and inactive tuberculosis	Sputum smear positive for AFB and positive culture for Mtb	English	MEIA	35.62 ± 9.48	26/16
12	Said [24]	Egypt	2013	22	1	5	19	27	34.60	Healthy individuals	Clinical, radiological and bacteriological findings	English	ELFA	36.5 (15–70)	14/13
13	Sun [25]	China	2019	37	197	6	449	43	22.00	Other respiratory disease	Clinical, radiological or bacteriological findings	Chinese	ECLIA	60.4 ± 22.1	−
14	Xu [26]	China	2013	29	12	15	66	44	30.25	Community acquired pneumonia	Clinical, radiological or bacteriological findings	Chinese	ECLIA	58	19/25
15	Yilmaz [6]	Turkey	2001	39	0	1	56	40	31.00	Heath individuals and inactive tuberculosis	Sputum smear positive for AFB and positive culture for Mtb	English	RIA	29.1 (16–60)	34/6
16	Yuan [27]	China	2015	26	5	34	27	60	35.00	Heath individuals	Clinical, radiological or bacteriological findings	Chinese	ECLIA	48 (18–72)	32/28

*TP* true positive; *FP* false positive; *FN* false negative; *TN* false negative; *ECLIA* Electrochemiluminescence immunoassay; *RIA* radioimmunoassay; *CMIA* chemoluminescent micro particle immunoassay; *ELISA* enzyme linked immunosorbent assay; *MEIA* microparticle enzyme immunoassay; *ELFA* enzyme linked fluorescent assay; *M/F* male/female; − no data

^*§*^Average age of initial treatment patients

^¶^Average age of retreatment patients

*Mean age and range of all participants

^#^M/F of all participants

### Quality assessment

**Fig. 2:**
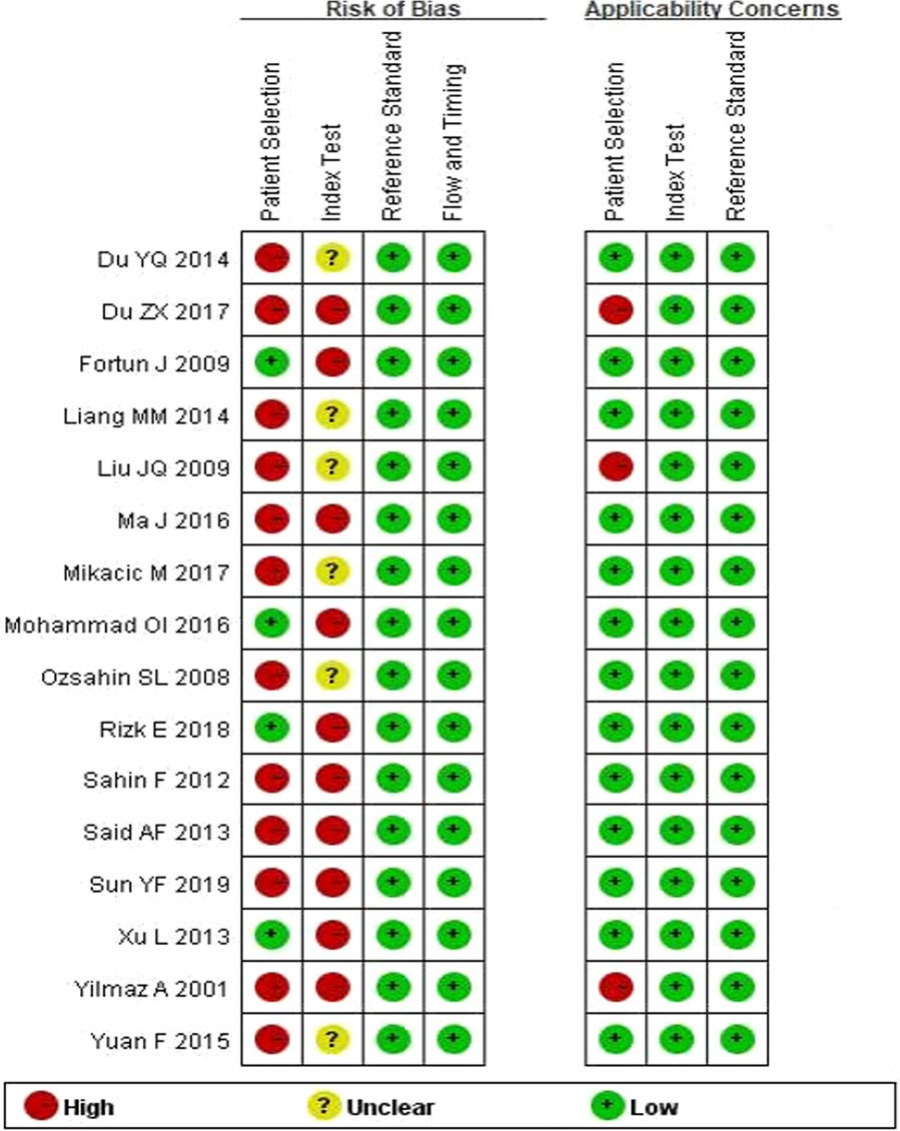
Summary of methodological quality of studies according to the QUADAS-2 tool

Two domains were identified as major risks for bias: patient selection and index test. Twelve studies indicated a high risk of bias in patient selection [6, 7, 19, 21–24, 26, 28–30, 32]. For example, whether random or consecutive samples of patients were enrolled was not clear in two studies [26, 32], the control groups did not include other respiratory diseases in eight studies [6, 7, 21–23, 28, 29, 32], and clear descriptions of whether the case groups excluded other combined diseases were not provided in seven studies [6, 7, 19, 22, 24, 26, 30]. Because none of the studies discussed whether the detection results of CA-125 were interpreted without knowledge of the results of the reference standard [6, 7, 19–32], these studies were likely to have unclear risks of bias in the index test. Thresholds were used in all eligible studies, but the corresponding values were not specified in 10 studies [6, 7, 20, 23, 25, 27–31]; thus, these studies were also likely to have a high risk of bias in the index test. Three studies had high applicability concerns in the patient selection domain [6, 7, 22] (Fig. 2).

### Summary estimates

**Fig. 3:**
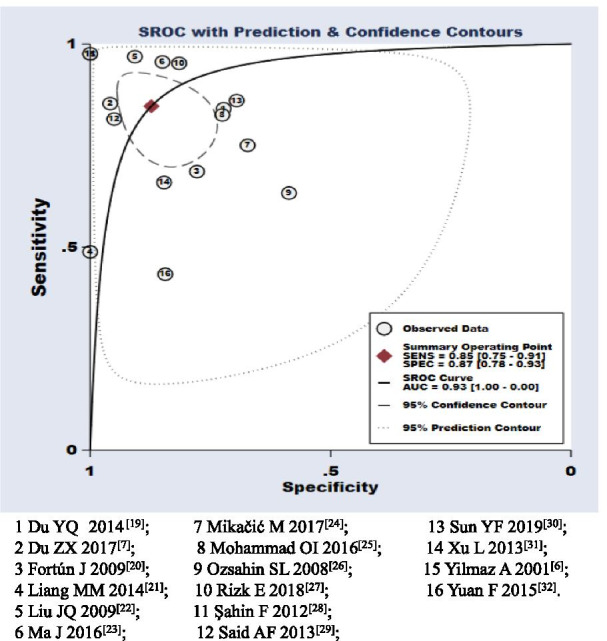
Summary receiver operating characteristics plot of sensitivity and specificity. Each circle represents an individual study; solid diamond in middle is summary sensitivity and specificity; inner ellipse represents 95% confidence region, and outer ellipse represents 95% prediction region

When all 16 studies were evaluated together, the pooled sensitivity, specificity, LR+, LR−, and DOR of CA-125 were 0.85 [95% confidence interval (CI) 0.75–0.91], 0.87 (95% CI 0.78–0.93), 6.65 (95% CI 3.62–12.20), 0.18 (95% CI 0.10–0.31), and 37.82 (95% CI 13.17–108.60), respectively (Table 3, Fig. 3). The area under the SROC curve (AUC) was 0.93. The SROC curve is displayed in Fig. 3.

**Table 3:** Pooled summary estimates of all studies

Accuracy measure	Pooled summary measure (95% CI)	Q value for heterogeneity	P value for heterogeneity	*I*^2^ value for heterogeneity (95% CI)
Sensitivity	0.85 (0.75–0.91)	181.74	< 0.001	91.75 (88.80–94.70)
Specificity	0.87 (0.78–0.93)	232.65	< 0.001	93.55 (91.41–95.69)
LR+	6.65 (3.62–12.20)	168.28	< 0.001	87.83 (87.83–94.35)
LR-	0.18 (0.10–0.31)	210.47	< 0.001	92.87 (90.44–95.31)
DOR	37.82 (13.17–108.60)	1.40E+14	< 0.001	100.00 (100.00–100.00)

*CI* confidence interval; *LR*+, positive likelihood ratio; *LR*− negative likelihood ratio; *DOR* diagnostic odds ratio

### Exploration of heterogeneity

The threshold effect is one of the main causes of heterogeneity in test accuracy studies. In the present meta-analysis, the “non-shoulder-arm” plot in the SROC space indicated no threshold effect (Fig. 3). In addition, the proportion of heterogeneity likely due to the threshold effect was 0.22; such a low value is indicative of the absence of a threshold effect.

The results of heterogeneity analysis for sensitivity, specificity, LR+, LR−, and DOR showed that all *P* values were less than 0.05 (Table 3), which indicates the presence of significant heterogeneity in the meta-analysis. All *I*^2^ values were greater than 75% (Table 3), which indicates strong heterogeneity. Meta-regression analysis revealed that “pre-specified threshold (Yes/No)” and “mean or median age ≥ 45 years (Yes/No)” were significant sources of heterogeneity in sensitivity (P = 0.01 and P < 0.001) and specificity (P = 0.04 and P = 0.03); specifically, the pooled sensitivity and specificity of studies in which these factors were answered “Yes” were lower than those of studies in which these factors were answered “No” (Table 4). “Control group includes other respiratory diseases (Yes/No)” was also a significant source of heterogeneity in specificity (P < 0.001), and the pooled specificity of studies in which this factor was answered “Yes” was lower than that of studies in which this factor was answered “No” (Table 4).

**Table 4:** Meta-regression analyses of sensitivity and specificity

Parameter	Category	Number of studies	Sensitivity (95% CI)	P1	Specificity (95% CI)	P2
Q1	Yes	14	0.87 (0.81–0.94)	0.08	0.89 (0.82–0.95)	0.36
	Uncertain	2	0.53 (0.18–0.88)		0.73 (0.42–1.00)	
Q2	Yes	8	0.80 (0.67–0.93)	0.06	**0.73 (0.64–0.82)**	< 0.001*
	No	8	0.88 (0.79–0.97)		**0.94 (0.90–0.98)**	
Q3	Yes	9	0.81 (0.69–0.94)	0.09	0.89 (0.81–0.97)	0.51
	No	7	0.88 (0.78–0.98)		0.84 (0.73–0.96)	
Q5	Yes	6	**0.74 (0.57–0.91)**	0.01*	**0.81 (0.67–0.96)**	0.04*
	No	10	**0.89 (0.82–0.96)**		**0.90 (0.83–0.97)**	
Q8	Yes	6	0.78 (0.62–0.94)	0.06	0.86 (0.75–0.98)	0.26
	Uncertain	10	0.88 (0.80–0.96)		0.88 (0.79–0.96)	
Country	Yes	8	0.82 (0.69–0.94)	0.11	0.87 (0.77–0.97)	0.24
	No	8	0.88 (0.78–0.97)		0.88 (0.78–0.97)	
Q11	Yes	5	0.87 (0.75–0.99)	0.61	0.87 (0.76–0.99)	0.39
	No	11	0.83 (0.73–0.94)		0.87 (0.78–0.96)	
Language	Yes	10	0.89 (0.81–0.96)	0.90	0.88 (0.80–0.97)	0.53
	No	6	0.76 (0.60–0.93)		0.85 (0.72–0.98)	
Method	Yes	8	0.82 (0.69–0.94)	0.11	0.87 (0.77–0.97)	0.24
	No	8	0.88 (0.78–0.97)		0.88 (0.78–0.97)	
M-age	Yes	10	**0.78 (0.66–0.90)**	< 0.001*	**0.83 (0.73–0.93)**	0.03*
	No	6	**0.92 (0.86–0.99)**		**0.92 (0.85–1.00)**	

Q1: Is a consecutive or random sample of patients enrolled? (Yes/No)

Q2: Dose the control group include other respiratory diseases? (Yes/No)

Q3: Dose the study include appropriate exclusions? (Yes/No)

Q5: If a threshold is used, is it pre-specified? (Yes/No)

Q8: Is there an appropriate interval between index test and reference standard? (Yes/No)

Q11: Is the number of cases >50? (Yes/No)

Country: China (Yes) or other country (No)

Language: English (Yes) and Chinese (No)

Method: ECLIA [Electrochemiluminescence immunoassay] (Yes) or other methods (No)

M-age: Mean or median age, ≥ 45 (Yes) or <45 (No)

*CI*: confidence interval; *: There are statistically significant differences in pooled sensitivity or specificity between the studies with “yes” and the studies with “no” in the items, suggesting that these items may be significant sources of heterogeneity in sensitivity or specificity

### Sensitivity analysis

**Fig. 4:**
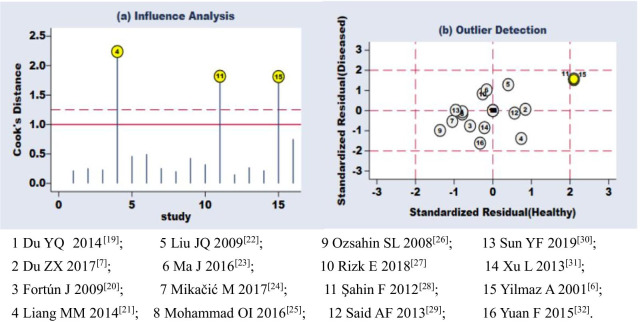
Sensitivity analysis

The results of sensitivity analysis revealed that the studies of Liang et al. [21], Şahin et al. [28], and Yilmaz et al. [6] were most influential in the present work (Fig. 4a). Among these studies, those of Şahin et al. [28] and Yilmaz et al. [6] were also identified as outliers with highly standardized residuals (Fig. 4b). When these three studies were excluded from the meta-analysis, the pooled sensitivity and specificity of CA-125 decreased from 0.85 to 0.83 and from 0.87 to 0.80, respectively.

### Publication bias

**Fig. 5:**
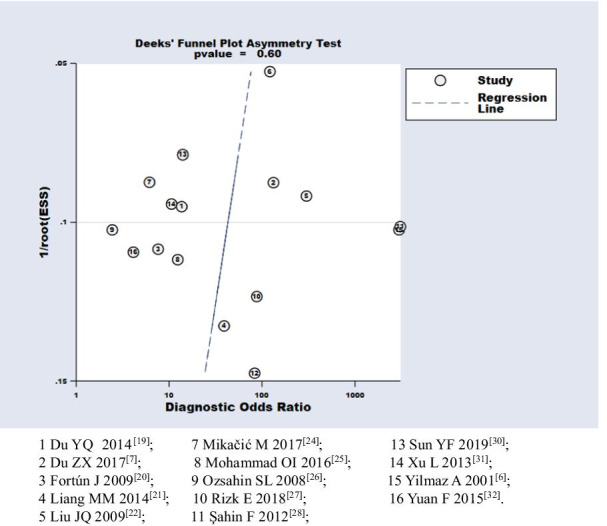
The potential publication bias assessment. The plot shows the symmetric distribution of the log of diagnostic odds ratios against the inverse root of effective sample sizes (ESS), indicating the absence of any publication bias

In the present study, Deek’s funnel plot asymmetry test did not reveal a striking publication bias (P = 0.60), and the funnel plot did not exhibit asymmetry (Fig. 5).

## Discussion

PTB can be confirmed by the presence of *M.tb* or its DNA in respiratory specimens. However, *M.tb* in respiratory specimens may not be detected in some cases of PTB, and respiratory specimens may not always be available; in this case, other detection methods, such as serological tests, must be used to assist in the diagnosis of PTB. CA-125 detection is a potential alternative detection method for PTB. CA-125 levels can increase in various malignant or non-malignant diseases. Previous studies suggested that serum CA-125 levels increase in patients with extrapulmonary TB [4, 33–35] and PTB [6, 7, 19–32]. However, to the best of our knowledge, the results of these studies have not been evaluated systematically.

A systematic review and meta-analysis of 16 studies were performed in the present work to assess the diagnostic performance of serum CA-125 in PTB patients. The results indicate that CA-125 detection could be beneficial in the diagnosis of PTB.

In addition to recruited studies, the studies that were excluded from the present meta-analysis due to lack of relevant information on sensitivity and specificity also suggested that serum CA-125 levels may assist in the diagnosis of active PTB and monitoring of therapeutic responses. A study in South Korea demonstrated that the mean serum CA-125 level (38.9 ± 41.4 U/ml) of patients with PTB is higher than the reference value (35 U/ml); however, only 38% of the patients in this study had serum CA-125 levels higher than the reference value [8]. The same study also suggested that elevated CA-125 levels are independently related to women, positive acid-fast staining of sputum, cavitary lung lesions, and involvement of more than one lung on chest X-ray, and CA-125 levels decreased after anti-TB treatment [8]. A study in Taiwan, China, also indicated that 45% of PTB patients had elevated serum CA-125 levels prior to treatment and that serum CA-125 levels decrease with improvements in anti-TB treatment outcomes [4]. Ichiki et al. found elevated serum CA-125 levels in 44.4% of patients with active PTB; after treatment with antituberculosis drugs, mean serum CA-125 levels significantly decreased [36]. Tascı et al. from Turkey observed that the serum CA-125 levels of PTB patients are significantly higher than those of healthy controls [37]. A significant decrease in serum CA-125 levels was observed after anti-TB treatment; however, if the serum CA-125 level was lower than 35 U/ml prior to treatment, the reduction achieved following anti-TB treatment was not significant [37]. The group also found that patients with a higher degree of sputum smear positivity have higher serum CA-125 levels [37].

Although previous studies suggested that serum CA-125 levels could assist in diagnosing active PTB and monitoring therapeutic responses [4, 6–8, 20, 21, 27–29, 32, 37, 38] and may be related to the severity of PTB [7, 8, 37], caution must be exercised when applying this finding to clinical practice. First, several studies have also reported that the proportion of PTB patients with elevated serum CA-125 levels is not especially high (38–45%) [4, 8, 36], which suggests that the serum CA-125 levels of most PTB patients may not be elevated. One study indicated that differences in CA-125 levels between the PTB and healthy control groups are not statistically significant [39]. Second, the recruited studies may present some risks for bias. For instance, the control groups in 8 studies did not include other respiratory diseases, 7 studies did not clearly describe whether the case group excluded other combined diseases, 10 studies did not provide specific thresholds, and all studies failed to illustrate whether the index test results were interpreted without knowledge of the results of the reference standard. Third, the recruited studies demonstrated significant heterogeneity. Meta-regression analysis indicated that the variables “control group includes other respiratory diseases,” “pre-specified threshold,” and “mean or median age ≥ 45 years” were associated with lower diagnostic specificity and/or sensitivity of CA-125 in the diagnosis of PTB.

Because CA-125 is present in mesothelial cells of the pleura, pericardium, or peritoneum, especially in areas of inflammation, increases in CA-125 are consistently observed in diseases involving these structures [9]. Serous effusions derived from these structures have been associated with increased serum concentrations of CA-125 [40–44]. Huang et al. suggested that the serum CA-125 levels of patients with tuberculous serositis (234.82 ± 279.25 U/ml) are significantly higher than those of PTB patients (48.26 ± 53.30 U/ml) [4]. Diabetes mellitus and adenocarcinoma may also increase serum CA-125 levels. Du et al. demonstrated that the serum CA-125 levels of PTB patients with type 2 DM (82.04 ± 82.96 U/ml) are significantly higher than those of PTB patients without DM (46.56 ± 42.47 U/ml) in initial treatment; moreover, the serum CA-125 levels of pulmonary adenocarcinoma patients (287.95 ± 341.64 U/ml) were also significantly higher than those of PTB patients with type 2 DM in initial treatment and retreatment, PTB patients without type 2 DM in initial treatment and retreatment, inactive PTB, bacterial pneumonia patients, patients with type 2 DM without PTB, and normal controls [7].

Given this background, if the control group does not include other respiratory diseases or the case group does not exclude other comorbid conditions, the specificity or sensitivity of CA-125 may be expected to increase. Knowledge of the reference standard is likely to influence the interpretation of the index test results [45], and the potential for bias could be associated with the subjectivity of interpreting the index test and order of testing [16]. In addition, if the test threshold used in the original study on the accuracy of the diagnostic test is the optimal result selected on the basis of sensitivity and/or specificity, then the test performance is also likely to be overestimated [16]. In the present study, a mean or median age of ≥ 45 years may be associated with the lower diagnostic sensitivity and specificity of CA-125, thus suggesting that serum CA-125 may have better diagnostic value in younger patients than in older ones. While only three studies were determined as most strongly influential in this meta-analysis and two of these studies were identified as outliers with high standardized residuals in the sensitivity determination, exclusion of these three studies did not remarkably affect the pooled sensitivity and specificity of CA-125. No striking publication bias was detected. These findings strengthen the validity of the results of our meta-analysis.

The present study presents several limitations. First, the meta-analysis did not recruit cohort studies, which may lead to overestimation of the test performance of serum CA-125. Second, the quality of the recruited studies was not high, and significant performance heterogeneity was noted; these limitations may affect the accuracy of serum CA-125 in the diagnosis of PTB. Although the results of the meta-regression analysis could explain part of the heterogeneity detected in the accuracy estimates, a considerable proportion of the heterogeneity observed remained unexplained. Third, although no major publication bias was found in this study, the possibility of publication bias cannot be completely excluded because positive results are generally more likely to be published than negative ones. Finally, although an extensive search for eligible sources was conducted, some qualified studies may still have been missed.

## Conclusions

In conclusion, CA-125 presents potential practical value for diagnosing PTB, but its clinical applicability must be further examined. The combination of CA-125 with other clinical information, such as clinical symptoms, chest radiography, microscopy screening, cultivation or molecular testing of *M.tb*, IGRA, and the tuberculin skin test (TST), is recommended in clinical practice. Large, multicenter, high-quality studies should also be conducted to strengthen the case for its use.

## Data Availability

The datasets used and/or analyzed during the current study are available from the corresponding author on reasonable request.
